# Using Telemedicine to Monitor the Patient with Chronic Respiratory Failure

**DOI:** 10.3390/life11111113

**Published:** 2021-10-20

**Authors:** Nicolino Ambrosino, Paola Pierucci

**Affiliations:** 1 Istituti Clinici Scientifici Maugeri IRCCS, Respiratory Rehabilitation Division of the Institute of Montescano, 27040 Montescano, Italy; 2 Cardiothoracic Department, Respiratory and Critical Care Unit, Bari Policlinic University Hospital, 70121 Bari, Italy; paola.pierucci@policlinico.ba.it; 3 Section of Respiratory Diseases, Department of Basic Medical Science Neuroscience and Sense Organs, University of Bari ‘Aldo Moro’, 70121 Bari, Italy

**Keywords:** COPD, e-health, ICT, long-term oxygen therapy, neuromuscular diseases, non-invasive ventilation, telemedicine, ventilator-assisted individuals

## Abstract

**Background**: Advances in management have improved mortality of individuals with chronic respiratory failure (CRF), leading to an increase in need for long-term oxygen therapy and/or ventilatory support. These individuals require frequent visits and monitoring of their physiological parameters as well as of the functioning of their devices, such as ventilators or oxygen concentrators. Telemedicine is a clinical application of Information Communication Technology connecting patients to specialised care consultants. This narrative review aims to explore the current available telemonitoring options for individuals with CRF and reported or potential results. **Methods**: The research focused on EMBASE, CINALH, PubMed, and Scopus databases. Papers published between 2003 and 2021 in English were considered. **Results**: Different sensors, transmission devices and systems, and interventions are used with promising but not conclusive clinical results. However, legal problems are still unsolved, and economic advantages for health care systems, although potentially high, are still under debate. **Conclusions**: Telemonitoring systems for individuals with CRF are increasingly used; with promising results still to be clarified, legal, economical and organisational issues must be defined.

## 1. Introduction

Advances in management have improved the mortality and morbidity of individuals with advanced chronic respiratory diseases, including those admitted to intensive care units for acute respiratory failure [1,2,3]. As a consequence, the prevalence of individuals with chronic respiratory failure (CRF) and/or needing long-term oxygen therapy (LTOT), non-invasive ventilation (NIV), or invasive ventilation (ventilator-assisted individuals (VAIs)) is increasing. This is due to prolonged survival of patients with advanced chronic respiratory diseases such as chronic obstructive pulmonary diseases (COPD), restrictive, thoracic, and neuromuscular diseases (NMD) [4,5,6,7].

Reported prevalences of individuals requiring home mechanical ventilation (HMV) vary among countries: from 6.6 to 23.0 per 100,000 people [8,9,10,11]. Long-term oxygen therapy is an accepted treatment for chronic hypoxemia of individuals with severe COPD and increasingly also for other end-stage respiratory diseases. Estimates suggest that in the United States, more than one million Medicare recipients receive LTOT with costs exceeding USD 2 billion dollars/year [12,13,14]. 

There are challenges when providing HMV and/or LTOT, including patient and caregiver training in the use of therapeutic tools, adequacy of care delivery, reimbursement policy, and patient (family) professional interaction [15]. Factors such as a natural history of chronic diseases including exacerbations and comorbidities, the need for technology, and a lack of professional supervision make the management of patients with CRF by families and non-professional caregivers a difficult task. In addition, the supervision by external companies has shown many limitations, such as a lack of standardisation, absence of regular feedback to the prescribing centers, high costs, and logistical problems [16]. Farré et al. [17] found discrepancies between prescribed and applied main ventilator settings. Chatwin. et al. [18] reported that ventilator alarms were not always available or set correctly for individuals using NIV, resulting in an increased workload of homecare providers. The need to reduce healthcare costs and to improve safety has prompted the development of new modalities of care for VAIs and other individuals suffering from CRF. 

Telemedicine consists of a clinical application connecting a patient with specialised care consultants, by means of electronic platforms and has been defined as ‘*Distribution of health services in conditions where distance is a critical factor, by health care providers that use information and communication technologies (ICT) to exchange information useful for diagnosis where doctor is able to perform diagnosis at distance*’ [19]. Increasing application of ICT to health care and advances in wearable and data transmission technology have led to telemedicine programs [20], potentially useful in improving the delivery of care as well as better patient compliance to chronic management [21]. In recent years, other similar terms have been used to define the role of ICT applied to health care systems, such as “telemonitoring”, “telecommunication”, “teleconsultation”, “telerehabilitation”, and “telecare” [22]. 

Telemonitoring consists of the recording and transmission of physiological and other non-invasive data and aims at reducing hospitalisations, improving self-care, and enhancing health-related quality of life (HRQL) [21]. This review explores the current available telemonitoring options for individuals with CRF.

## 2. Methods

### Literature Search

To perform this narrative review, a literature review was performed on the electronic platforms Medline, Pubmed, and Scopus for articles published in English from 2003 to 2021. The following keywords were used: “Chronic respiratory failure”, “Chronic respiratory diseases”, “Respiratory Failure”, and “Respiratory Insufficiency”. Each of these terms was then combined with: “telemedicine”, “Mobile health”, “telemonitoring”, “telecommunication”, “teleconsultation’, ‘telerehabilitation”, and “telehospice”. Finally, the references list of the retrieved articles was tracked down, looking for other relevant studies or reviews. No specific guideline was used, neither was any quality evaluation of papers performed. The review was not registered.

## 3. Results

### 3.1. Telemonitoring Options

Recent advances in sensors, miniaturised processors, body area networks, and wireless data transmission technologies allow the assessment of environmental, physical, and physiological parameters in different environments, without restriction of activity. Sensors may be grouped in categories such as respiratory monitors, pulse oximeters, activity trackers, environmental sensors, and monitors of other physiological variables [20] (Table 1).

Wearable sensors, smartphone applications (apps), and miniaturised processors are used in telemedicine. Body area networks (BANs) are systems composed of wearable devices attached in fixed positions or carried by the person. Wireless BANs perform measurements and transmit the obtained values via wireless communication allowing monitoring physical, physiological, and biochemical parameters in different environments at rest and during activities and interventions when a critical event is identified [20].

These devices can be used in different interventions such as [20,21]:

- Real time or ‘store and forward’ video;
- Telephone links to and from patients and caregivers;
- Internet-based telecommunication, digital;
- Broadband, satellite, wireless, or Bluetooth transmission to and from patients.

### 3.2. Application in Chronic Respiratory Failure

Patients with CRF are characterised by the failure of respiratory system in one or both of gas exchange functions: oxygenation and carbon dioxide (CO_2_) removal. They may require LTOT, NIV, or invasive ventilation at home.

#### 3.2.1. Long-Term Oxygen Therapy

Home oxygen therapy improves the prognosis of individuals with COPD and other CRF [23]. Telemonitoring data of the devices delivering oxygen such as oxygen concentrators or portable carriers, and of patients’ physiological and clinical conditions can be stored on a server and accessed remotely by health care providers on demand, enabling them to check the performance of and the compliance to LTOT and, in the case of need, to intervene appropriately. In countries such as Japan, LTOT telemonitoring is covered by social insurance systems and is recognised as a new medical technology [24].

#### 3.2.2. Home Mechanical Ventilation

Information communication technology may offer remote monitoring of physiological and clinical condition, as well as of ventilator setting and potentially remote control of ventilator including the remote monitoring of HMV initiation at home [21,25]. Depending on the used device, data monitoring can be continuous or on demand and, according to the information gathered, the professional can prescribe a change in ventilatory settings for the home care provider to be carried out at home or even potentially change settings directly via remote.

There are several parameters potentially to be monitored and transmitted to a central control [26]:

- Pressure curves to monitor effective setting delivered from the ventilator;
- Delivered tidal volumes and minute ventilation;
- Effective patient–ventilator interaction and synchrony;
- Combination of ventilation curves, pulse oximetry (SpO_2_), exhaled or transcutaneous carbon dioxide (TcCO_2_).

#### 3.2.3. Advantages and Problems

Options offered by telemonitoring to deliver LTOT and/or HMV may vary depending on the local available resources and personnel dedicated [27]. There is a growing body of literature which highlights the important advantages telemonitoring may offer to health care providers and patients:

- Reductions in number and costs of emergency department admissions and hospitalisations [27,28,29,30,31,32].
- Improvement in HRQL of patients and care givers [30,33,34,35].
- Reduction in the number of out-patient visits, reducing the burden (bringing their devices) of travel for individuals with disabilities and multiple comorbidities [30,36,37,38].

However, there are specific issues and potential obstacles to consider:

- No technology will be ever able to replace the face to face approach and the needed empathy between the doctor and the patient.
- Demographics (e.g., older individuals), education (low levels vs. more educated people), socio-economic conditions with access to internet, confidence in technology, cognitive, motor, visual, phonation and speech abilities, family and home environment may allow, prevent or influence the use of these technologies. Most individuals with CRF are older, often with physical or cognitive limitations. Therefore, they may face difficulties to approach the technical use of personal computers and/or to interact with technology equipment, requiring professional or non-professional support [39].
- Costs of equipment may be high for hospitals and patients.
- Fast internet connection is not yet available everywhere with potential audio and video difficulties during teleconsultations.
- Despite recent progresses, many countries are facing legal, privacy, data security, and economic issues without any precise dedicated rule or law yet [40,41,42].

### 3.3. Clinical Results

Non-invasive ventilation is widely used to treat chronic hypercapnia due to COPD and other diseases [5,7,8,9,10,11]. Clear benefits from NIV use for stable individuals with COPD and chronic hypercapnia are not well established [43]; however, outcomes may be improved with higher inspiratory pressure levels aimed at normalising arterial CO_2_ tension [44,45]. Because of relatively high utilisation of HMV, patient complexity, and the burden of needed services, health care systems are challenged to meet costs. To solve there may be a transitioning from higher-cost hospitals to lower-cost locations, such as home or institutions with less professional assistance. As a consequence, the majority of the burden for the management of HMV may fall on patients and families.

Telemedicine may provide some assistance with the transition, although 23% of patients under HMV may be resistant to remote monitoring [15]. Reflecting on the potential role for telemedicine in the application of HMV, a European Respiratory Society (ERS) Task Force developed an official statement [21]. Relevant issues included identifying the best care models, need for legal clarity with these therapies, and the lack of evidence of cost-effectiveness [21]. The Task Force concluded that while remote health monitoring systems and wearable sensors are available [20], evidence for their cost-effectiveness is still inconsistent [21].

A survey of HMV users (almost 800 patients and caregivers from 11 European countries) showed that about 50% of respondents indicated they would be confident with a telemonitoring system of their HMV program [15], which on the other hand, is currently an option only for 5% of patients with COPD under long-term NIV [46]. Another survey [47] showed that about one-third of responding community COPD services were using telehealth, believing it to be effective despite the lack of robust evidence in the literature. However, several problems were described in its use, for instance, the large variety of monitored variables, the increased number of hardware available, and the different techniques to set alarm limits, which, as a consequence, were responsible for false alarms [18,47].

Several chronic respiratory conditions with CRF may benefit from telemedicine.

#### 3.3.1. COPD

Chronic obstructive pulmonary disease has been studied more than other respiratory diseases. Vitacca et al. [30] showed that nurse-centred telecare reduced the hospitalisation rates of individuals with CRF, and was cost-effective, the greatest advantage being observed in individuals with COPD. These results have been confirmed in a follow-up study by the same authors [36], who found that the overall telemedicine costs were lower, the physician’s time dedicated to the program decreased, while the nurses’ time increased. They also confirmed that home monitoring of individuals on HMV was feasible, and useful for titration of oxygen, mechanical ventilation settings, and to avoid hospitalisations [38].

##### How to Initiate HMV

One specific issue is how to initiate long-term NIV and how telemedicine can help. Discharge of chronically critical patients may be a risky procedure and the clinician should have full control of the prescriptions. Modern home ventilators fulfil quality and safety criteria [16]; however, performance and applicability may not be so reliable. The type of circuit, accessories, and available modes of ventilation may greatly vary among ventilator machines. In addition, the same ventilators may show differences in triggering, rise time, pressurisation capacities, maximal flow provided, cycling and level of expiratory positive airway pressure. Therefore, there is the need to increase and spread knowledge regarding current ventilator machines available among all health care providers involved in their use. This would allow tailoring of the ventilator equipment prescription and the related telemonitoring systems [48].

Due to the increased number of individuals with COPD and CRF requiring NIV, recently the home initiation of NIV became a reality. Indeed, a randomised controlled trial (RCT) has shown that home initiation of long-term NIV with the use of telemonitoring in stable hypercapnic individuals with COPD is non-inferior to in-hospital initiation; it is safe and reduces costs by over 50% [29].

Therefore, telemonitoring can represent an efficient management of these chronic complex individuals.

##### Exacerbations

An interesting aspect of telemonitoring is the potential ability to “predict” exacerbations. There are studies showing significant changes in physiological variables before an exacerbation, thus providing a useful tool to be transmitted, allowing early interventions [20]. Physiological parameters such as heart rate, SpO_2_, peak flow, respiratory rate, alone or combined, have been used with variable results [49,50,51,52,53]. It has been shown [32] that in chronically hypercapnic individuals with COPD on LTOT, teleassistance alone and with greater efficacy when combined with NIV may reduce the exacerbation rate. However, a RCT has shown that telemonitoring added to standard care did not shorten the interval between the next acute hospital admission, nor increased hospital admissions or overall home visits, and did not improve HRQL in individuals with chronic respiratory conditions [28]. Another RCT, including elderly individuals with COPD from stage I to IV, showed similar results and did not find any significant difference in time to first hospitalisation nor in HRQL [54]. A systematic review suggested that adding telemonitoring to usual care reduced unnecessary emergency room visits but was unlikely to prevent hospitalisations due to COPD exacerbations. The same study also described that telemonitoring was well accepted by individuals with COPD and that it could be easily integrated into their existing care [55].

#### 3.3.2. Neuromuscular Diseases

There are many advantages from the use of telemedicine in NMD, such as amyotrophic lateral sclerosis (ALS), enhancing access to specialist care [56,57]. Individuals with ALS and CRF are chronically dependent on NIV or invasive ventilation, requiring frequent follow-up visits and multidisciplinary care [58]. The cost-effectiveness of telephone-accessed consultations with mechanical in-exsufflation and manually assisted coughing, and the oximetry feedback program, was evaluated in individuals with ALS [59]. The on-demand consult and mechanical in-exsufflation access program showed that it was feasible and could avoid hospitalisations with significant cost savings [59]. A few RCTs have recently highlighted that starting mechanical ventilation at home is cost effective, may improve HRQL, and is also not inferior to hospital initiation for individuals with ALS [5,30,37,60,61]. In other studies, remote initiation to HMV showed preserved quality and effectiveness with a reduced waiting time and improved survival [62,63]. However, the remote follow-up of individuals with ALS and CRF under HMV may be challenging. In this regard, some studies demonstrated that self-reported questions for telemonitoring together with weekly checks of pulse oximetry and ventilator settings may provide effective monitoring and may offer proactive support for optimised care [64]. Moreover, a long-term multidisciplinary telehealth- and telecare-integrated approach to individuals with CRF, including those with ALS, showed a reduction in new hospitalisation rates and costs and an increase in HRQL and patients’ satisfaction with the service [65,66]. Therefore, telemonitoring can represent an efficient management of chronic complex individuals suffering from NMD under HMV.

#### 3.3.3. Sleep Monitoring

The telemonitoring approach to individuals with hypoventilation syndromes under NIV follow-up is still unclear. Night monitoring is complex for many reasons, such as:

- Physiological variations of different variables;
- Clinical problems (pain and secretions, among others);
- Sleep disturbances.

Finally, almost all ventilator types are different, and knowing all the algorithms operating in the available machines in commerce is difficult, which may generate difficulties or even mistakes [48]. Marked differences can occur in ventilator performance, mode of triggering, pressurisation slope, and type of exhalation; in addition, leaks and changes in upper airway resistance may modify these patterns [21,67].

It has been reported that the use of home-based telemonitoring strategies for initiation of continuous positive airway pressure (CPAP) or NIV in selected individuals with obstructive sleep apneas (OSAS) is feasible, appears to be non-inferior to standard sleep laboratory procedures, and facilitates faster access to therapy [68]. However, the role of screening of OSAS before the initiation of home NIV is still discussed. Indeed, using an in-laboratory overnight polysomnography to titrate NIV in individuals with stable hypercapnic COPD who are initiating NIV is not recommended [69]. Therefore, there are perspectives for use of telemonitoring systems of a home-based prescription of NIV/CPAP.

In summary, different clinical results have been reported, and we need many more randomised controlled studies to evaluate the real effectiveness of this modality in clinical practice.

### 3.4. End of Life

The last disease stages of individuals with CRF are often difficult to manage in hospital and a strong integration between multidisciplinary teams is warranted including respiratory palliative care clinicians, specialist nurses, physiotherapists, psychologists and social workers. This kind of network proved to be effective to meet high quality standards for care of individuals with CRF [70,71], and many individuals with end stage respiratory diseases reported significant improvements in their HRQL and compliance with the disease [72,73].

However, this cannot be enough. With increasing awareness of the right to the relief of symptoms [71], the need for systems of care allowing home management will increase. In the literature, there are already studies which have evaluated the feasibility and effectiveness of remote monitoring of the end stages of disease and telehospice is the term coined to define the telecare of individuals during their end of life. A substantial body of research has been provided with the quality of the service available scored as very satisfactory by involved individuals [74]. However, despite the potential facilitation that ITC can provide to individuals in the end stages of their diseases and their families, many limitations are still to be faced. For instance, the virtual system may potentially induce increases in physical and psychological distance with carers and doctors [75].

### 3.5. Legal Issues

Telemedicine systems are complex, involving the interaction of cared people, caregivers, professional staff, and stakeholders. Legal issues are similar to conventional, face-to-face, doctor–patient relationships [76,77,78,79]. Specific problems may include failure to reach an acceptable standard of care, equipment/system failure, electronic data manipulation, failure of data protection, misunderstanding among patients, families and caregivers, and unclear responsibilities and obligations among health professionals. These issues must be addressed, balancing them against the potential benefits (Table 2).

From a legal perspective, the responsibilities require clear and complete documentation, including contracts, policies, and agreements. Even if the telemedicine systems can be considered as experimental, this refers only to their scientific, technical and economic aspects. Protection of personal data must be one of the most important issues. With respect to HMV, the ERS Task Force [21] recommended the promotion of common standards of practice for data transmission, management pathways, equipment, facilities, and regulations of telemonitoring systems.

### 3.6. Economics

Although a meta-analysis described a reduction in hospital costs and other savings [80], reports of cost-effectiveness are inconsistent and often obtained from poor-quality studies. As a consequence, decision-makers may have difficulties accepting telemedicine programs in health systems. In an acute care setting, a tele-ICU program with mobile platforms working with a rapid response team was able to deliver care support outside the ICU, reducing avoidable ICU admissions, without any adverse outcome and resulting in a 66% return on investment [81]. Optimal cost-effectiveness was achieved when tele-ICU was applied to the selected 30–40% highest risk ICU patients [82]. However, to evaluate the real cost-effectiveness of any new method of care such as telemonitoring, the definition of the compared ‘standard therapy’, should be specified in the frame of the different home care organisations. More cost-effectiveness studies are needed to provide a final answer because outcome measurements are too dependent on various factors rather than only technology and organisation.

### 3.7. Future Directions

The recent development of telemonitoring provides the possibility of adjusting ventilator settings remotely, based on the longitudinal assessment of NIV parameters and respiratory variables provided by the system. This may influence the future management strategies of health organisations for patients under home NIV [83].

The COVID-19 pandemic has pushed new developments in telemedicine. It is conceivable that in the near future, virtual reality, augmented reality, artificial intelligence, domotics, and robotics will relieve the burden of many facets of health care from professionals with more benefits and safety for more fragile individuals such as those suffering from CRF [20,84,85,86,87,88]. For instance, home automation systems may monitor and/or control functions such as lighting, climate, entertainment systems, but also other appliances such as control and alarm systems. The latter ones may also include clinical, physiological, and ventilatory parameters of patients and ventilators. Robotics might monitor patients’ beds, performing progressively more complex nursing tasks under at-distance human control. This would result in less physical burden on caregivers and even less potential risks of infection transmission.

Virtual reality has found application in rehabilitation programs [20,89,90]. New body area networks will allow development of signs and symptom monitoring and transmission [20,91,92]. An example was given in recent decades by studies exploring programs focused on breath sounds and breathing patterns to be transmitted to remote station using the internet for patients’ online monitoring [93]. This could be helpful, for instance, to check abnormal sounds production in individuals on HMV, e.g., crackles due to the onset of pulmonary oedema, or wheezing due to exacerbations of COPD. Moreover, signalling abnormal sounds may be useful during home NIV (e.g., masks leaks), or invasive ventilation (e.g., mucus plugs with abnormal sounds from the tracheal tubes). All these abnormal sounds through specific alarms setting and telemonitoring could be detected as anomalies and transmitted to the control/hub/hospital which can remotely provide guidance on how to resolve the related problem avoiding unnecessary emergency department visits or hospitalisations.

## 4. Conclusions

Telemedicine for patients with CRF has greatly improved in recent decades, thus allowing better care, safety, and greater satisfaction of cared people and caregivers. However, telemedicine is just one of the steps of the progressive intrusion of technology in the care of these patients. So far, no technology has ever been able to substitute the empathy of the in-person patient–caregiver relationship, and probably never will. Therefore, as health care providers, we always need to keep in mind the balance between the advances of available technology and the maintenance of empathy and a real approach to our most severely affected patients.

## Figures and Tables

**Table 1:** Sensors for telemonitoring of patients with chronic respiratory diseases [20].

Respiratory monitors	Spirometers
	Forced oscillation technique
	Continuous breathing monitoring Body area networks
Pulse Oximeters	
Activity trackers	Video based recognition systems Pedometers
Environmental monitors	
Other physiological variable monitors	Exhaled and Tc CO_2_ monitors Electrocardiographs Thermometers Arterial blood pressure
Abbreviation: Tc: Transcutaneous; CO_2_ carbon dioxide	

**Table 2:** Potential legal Issues.

Not appropriate standard of care for at distance consultation between patient/family and staff or among health care operators;
Failure of equipment or system with potentially lethal consequences;
Poor protection and/or manipulation of personal data;
Difficult distinction of responsibilities;
Potential obligations among each care-giver.
